# Stress Experience, Depression and Neck Disability in Patients with Temporomandibular Disorder—Myofascial Pain with Referral

**DOI:** 10.3390/jcm12051988

**Published:** 2023-03-02

**Authors:** Krzysztof Dariusz Szarejko, Maria Gołębiewska, Monika Lukomska-Szymanska, Joanna Kuć

**Affiliations:** 1 Private Health Care, Physical Therapy and Rehabilitation, Bialystok, 79 Warsaw St., 15-201 Bialystok, Poland; 2 Department of Dental Techniques, Medical University of Bialystok, 13 Washington St., 15-269 Bialystok, Poland; 3 Department of General Dentistry, Medical University of Lodz, 251 Pomorska St., 92-213 Lodz, Poland; 4 Department of Prosthodontics, Medical University of Bialystok, 24A M. Sklodowskiej-Curie St., 15-276 Bialystok, Poland

**Keywords:** temporomandibular disorders, myofascial pain with referral, orofacial pain, psychological factors, Perceived Stress Scale, Beck Depression Inventory, Neck Disability Index, TMDs, allostatic load

## Abstract

The etiology of temporomandibular disorders (TMDs) is firmly anchored in the biopsychosocial model in which a special role is attributed to the stress, depression, somatic symptoms, and anxiety. The aim of the study was to assess the level of stress, depression and neck disability in patients with temporomandibular disorder—myofascial pain with referral. The study group enrolled 50 people (37 women and 13 men) with complete natural dentition. All the patients underwent a clinical examination according to the Diagnostic Criteria for Temporomandibular Disorders and were diagnosed as individuals with myofascial pain with referral. The questionnaires were associated with stress, depression, and neck disability; Perceived Stress Scale (PSS−10), Beck Depression Inventory(BDI), and Neck Disability Index (NDI) were evaluated. Of the individuals evaluated, 78% showed elevated levels of stress, and the average value of the PSS−10 in the study group was 18 points (Me = 17). Furthermore, 30% of the subjects presented depressive symptoms, with the average value of BDI was 8.94 points (Me = 8), and 82% of the subjects showed neck disability. The multiple linear regression model revealed that BDI and NDI allowed explanations for the 53% differentiation of PSS−10. In conclusion, stress, depression, and neck disability coexist with temporomandibular disorder—myofascial pain with referral.

## 1. Introduction

Temporomandibular disorders (TMDs) are recognized as a group of specific conditions associated with masticatory muscles, temporomandibular joints, and associated structures [1]. TMDs are the second most common musculoskeletal disorder that cause pain and disability [2,3]. The most accepted model for TMDs classification and diagnosis is the Diagnostic Criteria for Temporomandibular Disorders (DC/TMD) [4]. According to axis I of this model, TMDs could be divided in Group I: Pain-Related TMD and Headache (myalgia, local myalgia, myofascial pain, myofascial pain with referral, arthralgia, headache attributed to TMD; Group II: Intra-articular joint disorders (disc displacement with reduction, disc displacement with reduction with intermittent locking, disc displacement without reduction with limited opening, disc displacement without reduction without limited opening); Group III: Degenerative Joint Disorder and Group IV: Subluxation [4].

Myofascial pain is an important severe socioeconomic public health problem [5]. It appears as a chronic, painful, and costly dysfunction affecting a significant part of the human population [6]. The prevalence of generalized myofascial pain is currently reported in 30–85%, while myofascial temporomandibular disorder is reported in 45.3% of cases [7,8,9]. The frequency variation observed in the literature results from different protocols and methods of testing.

The pathogenesis of myofascial pain remains still unclear, however the overload of a properly blood-supplied muscle, ischemia of working muscle, sympathetic responses leading to changes in the supply of vessels, and modified mental and emotional condition can be relevant factors [10]. People suffering from myofascial pain demonstrate depressive symptoms, decreased performance in activities of daily living, and reduced quality of life [10].

As a temporomandibular disorder, myofascial pain with referral is manifested through pain of a muscle origin, including pain spreading beyond the boundary of the masticatory muscles [11]. Secondary to pain, limitation in the range of mandibular motion appears. Within masticatory muscles develop taut bands identified as discrete foci of hypercontracted fibers [11].

The etiology of TMDs is firmly anchored in the biopsychosocial model. Currently, this is the most accepted concept which includes combination of overlapping biological, psychological, and social factors [12]. In this model, a special role is attributed to the coexistence of TMDs with stress, depression, somatic symptoms, and anxiety. These components affect TMDs treatment. Nowadays, a special role is attributed to psychological therapies including stress management, biofeedback, cognitive behavioral therapy, and relaxation [13]. There is some evidence showing that these therapies could be better than other methods of reducing psychological distress [14]. Symptomatic treatment of TMDs involves occlusal splint therapy, pharmacotherapy, physiotherapy, occlusal equilibration, orthodontics, and prosthodontic treatment [8,15]. In recent years, scientists have more attentively studied the attraction of interdependence of TMDs with headaches, irritable bowel syndrome, lower back pain, and fibromyalgia. All of these coexist under a common term—Chronic Overlapping Pain Condition (COPC) [16,17].

As above mentioned, special factors of interest in the etiology of TMDs are stress and depression. Stress is defined as a multifactorial complex phenomenon in which basal organic resources are mobilized to deal with a real or potential threat. The reaction to stress is recognized as the one of the major phylogenetic coping mechanisms that enables people to adapt successfully to very challenging and dangerous tasks [12,18]. Nowadays, the neurobiological mechanism controlling the stress response (hypothalamic-pituitary-adrenocortical axis and autonomic nervous system) does not keep up with the changing world [18]. People experience dissonances between adaptive psychophysiological stress reactions to physical and psychosocial stressors, as well as chronic stressors of current Western culture [18]. The consequence is the activation of neuroplasticity in central stress-processing networks, thereby causing sensitization and habituation of the hypothalamic-pituitary-adrenocortical axis (HPA) and autonomic nervous system. It results in serious health implications [18,19].

In turn, depression is defined as a mood disorder characterized by a persistent feeling of sadness and/or loss of interest [20]. This condition represents change from the previous level of human functioning in which at least one of the symptoms must be depressed mood or lack of pleasure. Typical features of depression are decreased interest in activities, weight loss or weight gain, eating disorders, insomnia or hypersomnia, excessive or reduced psychomotor activity, fatigue, lack of energy, impaired sense of value, excessive guilt, impaired ability to think and to concentrate, lack of determination, death thoughts, and suicidal thoughts or suicide attempts. However, the symptoms cannot be related to bereavement, medical disorders, or certain substances [20]. All symptoms have been divided into emotional, cognitive, motivational, physical and vegetative, and delusional categories [20,21].

Stress and depression seem to be mediators between pain and disability [22]. Both of them are important risk factors for neck pain [22]. They can modulate central pain processing in the spinal cord, brainstem, and cerebral cortex which may manifest as hyperalgesia—a state in which people experience an aggravated sensitivity to pain [22,23,24,25]. The suitable example of central sensitization could be the coexistence of migraine and TMDs resulting in worse levels of hyperalgesia and cutaneous allodynia [26]. This interdependence may contribute to a vicious circle in TMDs patients, and in many cases, lead consistently to furthering functional limitations including smiling, talking, or yawning [27]. Due to the fact that TMDs have overlapping nature with other conditions such as fibromyalgia, headache, and neck pain, in each case of TMDs, the central pain sensitization should be considered. The treatment must be multidisciplinary. Sometimes, it should include therapies targeted at central pain sensitization [28,29].

The aim of the study was to assess the level of stress, depression, and neck disability in patients with temporomandibular disorder—myofascial pain with referral.

The hypothesis was that there are gender-related differences in prevalence of depression, neck disability, and the severity of stress. Bearing in mind that temporomandibular disorders are of multifactorial nature, it was hypothesized that at least one of the variables such as depression and neck disability has a statistically significant relationship with stress in people with myofascial pain with referral. It was also considered that there is a statistically significant prediction of stress by depression and neck disability.

## 2. Materials and Methods

### 2.1. Study Design

The present study was performed after obtaining the consent of the Bioethics Committee of the Medical University of Bialystok, Poland (decision No. R-I-002/322/2016). The research was conducted according to the principles of the Declaration of Helsinki of the World Medical Association (WMA) as well as the Guidelines for Good Clinical Practice (GCP). Participation in the study was voluntary. Comprehensive information about the nature, scope of clinical activities, and the course of the proceedings were presented to all study participants. At any time and without any resulting consequences, the patients had the right to withdraw their consent to participate in the study. Written consent was obtained from each participant.

This pilot study comprised 50 people (37 females and 13 males) with complete natural dentition, who had been referred to the Department of Prosthodontics at the Medical University of Bialystok, Poland, from September 2016 to November 2017. All the patients underwent a clinical examination according to the Diagnostic Criteria for Temporomandibular Disorders (DC/TMDs) [30]. The patients were individually diagnosed with myofascial pain with referral (Axis I of DC/TMDs) [30]. Clinical examination of the patients and screening for inclusion and exclusion criteria by asking related questions were performed by researcher who was also a dentist and physiotherapist (the author J.K.).

*Inclusion criteria*:

- Myofascial pain with referral with respect to I axis of DC/TMD;
- Self-reported pain of at least 8 points according to VAS (Visual Analogue Scale) within craniofacial and/or craniomandibular area;
- Complete natural dentition (Angle’s Molar Classification and Canine Position—Class I);
- Lack of orthodontic history or retention status over 3 years after the completion of treatment.

*Exclusion criteria*:

- Any craniofacial and/or craniomandibular trauma (e.g., fractures, whiplash injury);
- Surgical treatment within the craniofacial and/or craniomandibular area (e.g., any orthognathic surgery, third molar extraction within the last year before examination, any previous cancer treatments, any botulinum toxin injection for masticatory muscles, any arthrocentesis of temporomandibular joint);
- Any occlusal splint therapy;
- Any prosthetic treatment;
- Any physiotherapy within the craniofacial and/or craniomandibular region;
- Any diseases which possible health concerns could affect the functioning of the masticatory muscles (e.g., hypothyroidism and hyperthyroidism, epilepsy, myopathy, muscular dystrophy, amyotrophic lateral sclerosis, multiple sclerosis);
- Any metabolic diseases (e.g., diabetes, osteopenia/osteoporosis, disorders of calcium and phosphorus metabolism);
- Chronic medication intake (in the past and at present).

The abovementioned strictly defined inclusion and exclusion criteria were determined by the whole expanded study design based on one concise group of the patients who suffered from myofascial pain with referral [27,31,32,33,34]. It should be emphasized that occurrence of any of the exclusion criteria could significantly affect the modulation of the biopsychosocial profile including the development of mechanisms of central pain sensitization. In the presented study, it was unacceptable.

### 2.2. Questionnaires

The Polish version of the following questionnaires associated with stress, depression, and neck disability were evaluated:

- Perceived Stress Scale (PSS−10);
- Beck Depression Inventory (BDI);
- Neck Disability Index (NDI).

The PSS−10 questionnaire comprises 10 questions referring to daily stress over the last month. Each item is evaluated on five-point scale ranging from ‘never’ to ‘very often’ (never = 0, almost never = 1, sometimes = 2, fairly often = 3, very often = 4). Before calculating the general value of PSS−10, the score of items 4, 5, 7, and 8 should first be reversed (i.e., 0→4, 1→3, 2 = 2, 3→1, 4→0). The total score of scale is the sum of all points ranging from 0–40 [35].

The BDI questionnaire consists of 21 items concerning the psychoemotional condition experienced in the last 14 days. The response variants assigned to each question correspond to the gradually increased severity of symptoms. Each item is scored on a four-point scale ranging from 0 to 3. The level of depression is estimated by summing up all values. The total score can range from 0 to 63 points [36].

The NDI is the most frequently used tool for cervical related disabilities. It determines activity limitations due to neck pain. The NDI questionnaire comprises 10 questions concerning pain intensity, personal care, lifting, work, headaches, concentration difficulties, sleeping, driving, reading, and recreational activities [37]. The response to each question is scored on six-point scale with a possible 0–5 value. For each question, only one answer is marked. The total score obtained after summing up the individual numerical values for each question can range from 0 to 50. Larger values determine higher levels of self-reported neck disability. To report the result as a percentage, the final value of the score should be multiplied by two [37].

### 2.3. Statistical Analysis

A statistical analysis was performed using the Statsoft Statistica 13.1 software (Statsoft, Inc., Cracow, Poland), G Power v. 3.1.9.4 (G Power, Aichach, Germany) and PQ Stat v. 1.8.2 (PQ Stat Software, Poznań, Poland). The arithmetic mean, median, and measures of differentiation involving standard deviation were calculated. The Mann–Whitney U test was applied to assess significant differences in the groups divided in terms of gender. A Chi-square test with Yates correction for a 2 × 2 contingency table was applied to compare categorical variables. A one-sided Fisher’s exact test was applied in the case of small sample size, when the expected number of frequencies was below 5. All differences with *p* < 0.05 were considered statistically significant. A post hoc power analysis was performed for the one-sided Fisher’s exact test. The determination of the effect size, α and sample size (n) allowed us to assess the statistical power (1 − β). A multiple linear regression model for PSS−10 estimation was developed by selecting the variables that contributed significantly to the PSS−10. The sample size was calculated by using a priori power analysis for a one-sided exact test for linear multiple regression: random model (H0 R^2^ = 0, H1 R^2^ = 0.2, α = 0.05, 1 − β *=* 0.8). With two independent variables (predictors), α = 0.05, 1 − β = 0.8, a sample of 45 was sufficient to detect values of R^2^ ≥ 0.2.

## 3. Results

This study comprised 50 people—37 females and 13 males. The moderate level of stress was found in 33 (66%) of the patients, including 24 (65%) women and 9 (69%) men (Table 1). High severity was observed in 6 (12%) subjects, including 5 (14%) women and 1 man (8%). Moreover, eleven (22%) patients declared low levels of stress. There were no statistically significant differences in the frequency of elevated levels of stress with regard to gender (*p* > 0.05) (Table 1).

The average value of the PSS−10 in the study group was 18 points (Me = 17) (Table 2). In the group of females, this coefficient was 18.24 points (Me = 17). In the case of males, this parameter was slightly lower and amounted up to 17.31 (Me = 19) (Table 2). No statistically significant differences were observed with respect to gender (*p* > 0.05) (Table 2).

In the present study, fifteen (30%) subjects presented depressive symptoms (Table 3). In nine (18%) patients, mild mood disorders were noted. In the case of three individuals (6%), borderline clinical depression was reported. Moderate depression was described in the cases of three (6%) patients. No extreme or severe depression was noted in this study group. There were no statistically significant differences in the prevalence of increased level of depressive symptoms with respect to gender (*p* > 0.05) (Table 3).

The average BDI value in the study group was 8.94 points (Me = 8) (Table 4). In the case of females, this parameter was 8.68 points (Me = 8). In the group of males, this variable amounted to 9.69 points (Me = 8) (Table 4). No statistically significant differences of BDI were reported with regard to gender (*p* > 0.05) (Table 4).

Thirty (60%) subjects showed the presence of mild disorders related to the cervical part of the spine (Table 5). Moderate ailments were observed in ten (20%) individuals, including eight women (22%) and two men (15%). Severe disability concerned one person (2% of the respondents). There was no complete dysfunction in the group. In the case of nine (18%) individuals, no cervical disability was noted (Table 5). There were no statistically significant differences in the prevalence of increased level of neck disability with respect to gender (*p* > 0.05) (Table 5).

The average value of the NDI parameter was 10.16 points in the study group (Table 6). In females, this coefficient amounted to 9.57 (Me = 9), while in the group of males it was 11.85 (Me = 12) (Table 6). No statistically significant differences of NDI were reported with regard to gender (*p* > 0.05) (Table 6).

The multiple linear regression model revealed that BDI and NDI enabled the differentiation of about 53% PSS−10 cases (R^2^ = 0.531), and the prediction model was significantly better than the random one [F(2,47) = 26.600; *p* < 0.00], as in the former the average error in evaluating the level of PSS−10 was SE = 4.416 (Table 7).

**Figure 1:**
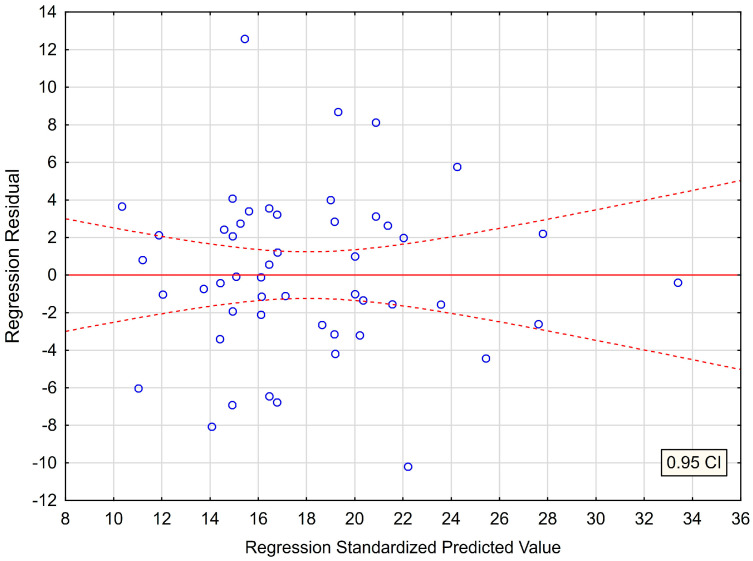
The plot of standardized residues versus standardized predicted values. (homoscedasticity) with respect to multiple regression model for PSS−10.

In the submitted regression model, the first assumption about linear relationship between predictor variables (BDI, NDI) and outcome variable (PSS−10) was fulfilled. The multiple regression equation was statistically significant [F(2,47) = 26.600; *p* < 0.00; R = 0.72865563] (Table 7). The next assumption about statistical significance of partial regression coefficients of BDI and NDI was also met (*p* = 0.00) (Table 7). The third assumption concerning the lack of multicollinearity (redundancy) between independent variables was fulfilled. This was confirmed by the obtained tolerance scores (BDI = 0.799069, NDI = 0.799069) and R^2^ values (BDI = 0.200931, NDI = 0.200931) (Table 7). Semipartial correlations confirmed low relationship between BDI and NDI with PSS−10 (r = 0.456844, r = 0.302649, respectively) (Table 7). The plot of standardized residues versus standardized predicted values revealed no obvious signs of a funnel suggesting that the variance of the residuals is constant (the fourth assumption about homoscedasticity) (Figure 1).

The fifth assumption about the lack of residual autocorrelation was also met. The Durbin–Watson statistic was close to 2.0 (Durbin–Watson = 2.010225) (Table 7).

**Figure 2:**
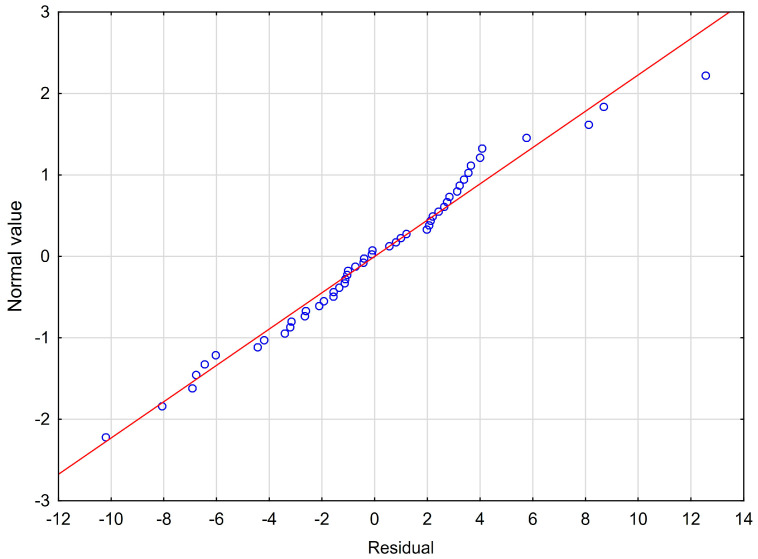
Normality of the distribution of residuals with respect to multiple linear regression model for PSS−10 estimation.

The next sixth assumption about the normality of the distribution of residuals could be violated (Figure 2).

Considering the fact that only extreme deviations from normality could have a significant impact on the findings, these study results are still valid. The seventh assumption about the lack of influential cases biasing the regression model was met. All of Cook’s distance values were below 1.0. This suggests that individual cases did not have an excessive effect on the whole model.

## 4. Discussion

The results of this study revealed an increased level of stress in people with temporomandibular disorder—myofascial pain with referral. The average value of the PSS−10 in the study group amounted up to 18 ± 6.31 points. A total of 66% of the patients had a moderate level, and 12% a high level of stress. Low severity was noted in 22% individuals (Table 1 and Table 2).

Dawson et al. showed that in the group of the patients with myofascial temporomandibular disorders, the PSS−10 score amounted up to 16.6 ± 5.9. In the control group, this parameter was at the level of 12.1 ± 3.6 points [38]. A total of 30 subjects were included in this research. In turn, Salameh et al. reported that in the study group of 60 people with temporomandibular dysfunction who lived in Damascus, Syria, the PSS−10 index was at the level of 23.98 points. In a similarly large control group, this score was 17.80 [39]. In the control and study group, the high level of stress was probably conditioned by cultural factors, and certainly by the political and economic situation. Salameh et al. also indicated that 70% of people suffering from pain were women [39]. Zieliński et al. reported that in female medical students PSS−10 score was connected with changes in muscular asymmetry in functional activity during teeth clenching [40]. These authors revealed the lack of statistically significant relationship between PSS−10 and activity of masticatory muscles during resting, teeth clenching with and without dental cotton rollers and on maximal opening of the mouth [40].

A study on a group of 201 generally healthy subjects (103 women and 98 men) aged ± 19 years indicated that only 30% of people suffered from a low level of stress [41]. Moderate and high levels of stress were noted in 40% and 30% of cases, respectively [41]. This demonstrates that the stress profile in the group of healthy subjects is closely related with the group of individuals affected by temporomandibular joint disorder—myofascial pain with referral presented in the present study.

Stress reflects disturbed homeostasis of the body and lack of harmony in its functioning. This condition is recognized as a ‘cycle of physiopsychic reactions to stimuli of danger, threat, obstacle, risk, challenge, novelty, excitement, and opportunity’ [12]. Long lasting stress leads to collective effects—cumulative trauma—on physical and mental health. This phenomenon is associated with allostatic load which refers to the long-term physiologic response to stress [42]. According to McEwen, allostasis is essential and even critical to survival [42]. It is recognized as the ability to achieve stability through change [42]. This is an adaptive mechanism that enables people dealing with stressors through physiological and behavioral processes [43,44,45]. Mc Ewen distinguished four types of allostatic load [42]. The first is considered as the frequently recurring stressful experience [42]. The second type means that there exists no adaptation to the same repeated triggers of stress. The consequence is prolonged exposure to stress hormones [42]. The next allostatic load includes inability to calm down allostatic responses when the stress is over [42]. In the fourth type, inappropriate reactions of allostatic system trigger the activation of compensatory mechanisms [42]. In this type of allostatic load, the impaired activity of one system does not ensure counter-regulation [42,44]. The allostatic load reflects measurement of long lasting stress which undermines physical and mental well-being. It occurs over the activated condition of hypothalamic-pituitary-adrenal axis and indicates dysfunction of multiple physiological systems [46]. This overload accelerates the biological wear and tear and aging. Among others, it favors medical conditions such as diabetes, obesity, cardiovascular and cerebrovascular diseases, depression, Alzheimer’s disease, and sleep disturbances [47,48,49,50].

It seems to be a major gap in the literature concern allostatic load and TMDs. The second axis of DC/TMD is a kind of ‘biopsychosocial biomarker’ of human well-being. However, to some extent, it limits the undertaking of causal treatment. For example, when a very high level of stress in TMDs patients is stated, an individual can be referred to learn skills and the mechanisms of stress management [8]. However, upon analysis of the issues of stress and associated allostatic load, it may occur that the patient requires a multifaceted lifestyle changes. Among others, these could concern alcohol consumption, diet quality, weight change, smoking status, physical activity, and sedentary behaviors [44]. It may be necessary to cooperate with many medical specialists, starting with primary care physicians. It is especially important in cases where the identification of the triggers of chronic pain is of paramount importance. The fact that physical activity is one of the factors reducing the level of stress and slowing cognitive aging and neurodegeneration may be an incentive [51,52,53]. The benefit of physical activity is increased plasticity and decreased inflammation in the hippocampus with all other consequences in the whole body [51].

As above mentioned, there is a necessity to incorporate allostatic load index as a gold standard in the clinical examination of TMDs patients. This index is based on four main biological systems—cardiovascular, metabolic, immune/inflammatory, and neuroendocrine [44,54]. The allostatic load is calculated by assaying and summing dysregulated biomarkers, which are determined using clinical or distributional thresholds [48]. A high score of allostatic load index refers to stress-related dysfunction reflecting poor long-term health outcomes [48]. Therefore, further research and clinical protocols with regard to allostatic load in TMDs patients are needed. This suggestion can be confirmed by a special type of allostatic load such as post-traumatic stress disorder (PTSD), which is often overlooked and underestimated in TMDs patients [55,56]. PTSD concerns mental health condition in which people, after having experienced a serious or threatening event, develop intrusive memories of the trauma, avoidant behavior, negative changes in cognition and mood, and changes in arousal and reactivity for four weeks or more [56]. Chronic painful TMDs and PTSD are known to be more common than would be expected based on the epidemiology of either state in the general population [56]. It should be emphasized that the prevalence of PTSD in people with painful TMD is currently reported from 15% to 24%, or even 31% [56].

Chronic stress is a major risk factor for depression which can cause cognitive changes persisting in individuals even though their mood has improved [57,58]. Depression and its progression precede acceleration age-related cognitive decline in the elderly [57,59,60].

These study results revealed a lower frequency of people suffering from depressive disorders in relation to the Beck Depression Inventory (BDI) than with respect to the PHQ-4 and PHQ-9 questionnaires which were presented in the previous publication [27]. In the case of BDI, only 30% of the respondents showed depressive symptoms of varying severity (Table 3). A total of 42% of individuals demonstrated health problems according to PHQ-4. With respect to the PHQ-9, 56% of the patients had symptoms of depression of varying severity [27]. This discrepancy probably results from the emotional, graduated character of the BDI questionnaire, which suggests a psychological assessment to the examined person, and enables the prediction of the expected outcome. In the case of PHQ-4 and PHQ-9, individual questions do not violate the respondent’s emotional comfort zone.

In the development of depression, a significant role is attributed to personality which is defined as a ‘complex of psychological properties that affect the characteristic patterns of human behavior, invariably in time and situation’ [61,62]. Special attention is focused on Type D personalities that reflect the equivalent of neurotic introversion [61]. Gebska et al. revealed that people with Type D personalities experienced significantly more TMDs symptoms [61], and tend to be more depressed [61]. The frequency of depression in Type D personalities amounted to 86.7%, while frequency in the control group was 20%. The same authors confirmed that individuals with depression are more likely to suffer from headaches, neck and shoulder girdle pain, TMJ acoustic symptoms, elevated masticatory muscle tension, and tooth clenching and grinding [61]. Moreover, patients with depressive symptoms have an increased risk of TMDs joint pain during palpation [63]. Consequently, a Type D personality may be recognized as a one of the TMDs predictors [61]. Interestingly, in people with depression, pain management is generally less effective [61].

Stocka et al. reported the symptoms of mild or medium depression in 18.82% of young adults. They revealed significantly higher activity of masticatory muscles in maximal voluntary clench in individuals with depressive symptoms in comparison to those without symptoms [64]. In turn, Zieliński et al. noted a lack of statistically significant influence of moderate depression on the bioelectrical resting activity of temporal and masseter muscles [65].

The presence of depression is often associated with an insufficient amount of vitamin D, which in turn could contribute to the occurrence of musculoskeletal disorders manifested by poor muscles strength and musculoskeletal pain [66,67]. Vitamin D probably determines the intensity of pain and pain management [67]. It is of particular importance with regard to the TMDs involvement in the potential mechanisms of central pain regulation [67]. It has already shown that in TMDs patients, combined treatment including supplementation of Vitamin D and occlusal splint therapy improved VAS score and the range of spontaneous and maximum mouth opening [67,68]. Interestingly, the Vitamin D receptor gene is considered as one of the most relevant candidate genes to contribute to TMDs [67]. Considering the fact that approximately one billion people worldwide may have Vitamin D deficiency and that numerous people with TMDs suffer from depression, evaluation of Vitamin D levels and individually tailored treatment may be required [66,69]. This may have an impact on the modulation of biopsychosocial profile and seems to be the challenge of further research.

Depression symptoms are inversely and significantly related to muscular strength [70]. General weakness appears as a consequence of reduced physical activity and associated functional limitations and disabilities [70]. As a one of the many possible clinical indicators for assessing muscle strength in depression, hand grip may be considered [70]. Regular physical activity improves muscular strength which is positively related to physical function [70]. It contributes to a decrease in symptoms of depression, which is the greatest non-communicable disease responsible for the loss of human well-being [70].

A special role in the etiology of painful TMDs may play neck disability and neuronal convergence. There is a neurophysiological relationship between orofacial and cervical areas through the trigeminocervical nucleus [71,72]. Nociception that develops from muscles and joints innervated by the upper cervical spinal nerves may be involved in overlapping ailments of other regions supplied by the trigeminal nerve [71,73]. Analogously, masticatory motor activity responsible for mandibular motion during talking, chewing, or mouth opening remains in relation with neck muscle activity [71,74,75]. Due to the neurological and biomechanical interactions, the cranium, mandible, and cervical spine create a functional complex that could be defined as ‘craniocervical mandibular system’ [15]. In this system, the position of head and neck seems to be the equilibrium of the cranium and cervical spine [15].

Von Piekartz et al. showed that in subjects suffering from an acute and subacute phase of TMDs, there are significantly more cervical impairments than in the control group [76]. In turn, Olivo et al. emphasized a strong relationship between the jaw functional limitation and neck disability index (NDI) [77]. These authors noted a strong correlation between the NDI coefficient and the chronic pain scale—GCPS—and the intensity of pain—CPI (chronic pain intensity) [77]. Winocur et al. highlighted that neck pain (prevalence of 46.6%) is the most frequent symptom connected with masticatory dysfunction in patients suffering from bruxism. This ailment is followed by orofacial pain and joint noises (28.2%; 21.2%, respectively) [78].

The results of present study showed that in the group of the patients with temporomandibular disorder—myofascial pain with referral, neck disability appeared. The NDI score was at the level of 10.16 ± 6.27 points. A total of 60% of the patients suffered from mild disorders, and in 20% of the subjects, a moderate level of dysfunction was found. A total of 2% individuals declared severe dysfunction. Only 18% of the subjects reported lack of cervical disability (Table 5 and Table 6). Gil-Martinez et al. reported no difference in NDI scores between patients with chronic migraine and persistent TMDs. The average values of NDI were 21.00 ± 8.18 and 19.92 ± 3.77, respectively [79].

According to the regression model, the present study reveals new findings involving stress, depression, and neck disability. NDI and BDI are covariates when PSS−10 is a criterion variable, explaining 53% of the variance in patients with temporomandibular disorder—myofascial pain with referral. It means that both NDI and BDI should be considered as a good predictive factor in the evaluation and management of people with TMD—myofascial pain with referral. It confirms that there is a necessity to assess the allostatic load in people with TMD. It also suggests that the upper cervical spine seems to be an important treatment target in patients with TMDs [79]. In conclusion, depression and neck disability are essential in understanding the stress in individuals with temporomandibular disorder—myofascial pain with referral.

### Strengths and Limitations of the Study

The presented study was conducted on a homogeneous group of patients diagnosed to strictly defined research criteria—DC/TMDs—as people with myofascial pain with referral (Axis I of DC/TMD). The use of the DC/TMDs protocol makes it easier for scientists to properly manage temporomandibular disorder and continuously develop the biopsychosocial model.

It is probably the first study that draws attention to the importance of stress in TMD, in the context of allostatic load as well as the first research that stresses on the need for mandatory marking of ‘biomarkers of allostatic load’ in patients with temporomandibular disorder—myofascial pain with referral. Considering the interdependence of TMD with cervical spine disorders as well as the results in relation to NDI, it may be necessary to require physiotherapy in this group of patients.

The first limitation of the study was the small sample size. The number of individuals in groups divided in terms of gender was imbalanced. Additionally, there was a risk of bias concerning selection, response, and confounding factors. Therefore, presented results should be treated with caution. Selection bias is usually determined by the study design and/or data collection. Response bias is associated with tendencies of participants to respond inaccurately or falsely to questions. This type of bias applies to all self-reported questionnaires. Confounding bias reflects a relationship which in fact does not exist or masks a true relationship.

Summarizing, the management of the biopsychosocial model requires interdisciplinary approach. TMDs management should be focused on biopsychosocial patient-centered screening and assessment. In many cases, the necessity of this type of cooperation may be a limitation.

## 5. Conclusions

Based on the results of the present study, it can be concluded that:

1. Stress and depression, as well as neck disability coexist with temporomandibular disorder—myofascial pain with referral. Depression and neck disability appear to be significant predictors of perceived stress levels.
2. The hypothesis that there are gender-related differences in prevalence of depression, neck disability, and the severity of stress has not been confirmed. Due to the small sample size, this cannot be excluded.
3. In order to complete the biopsychosocial model of temporomandibular disorders, further studies on the detailed assessment of allostatic load as well as cervical spine disorders are necessary.

## Figures and Tables

**Table 1:** Perceived Stress Scale with respect to the PSS−10. Numerical and percentage values are given for the study group (*n* = 50) and for the group of females (*n* = 37) and males (*n* = 13).

					Comparison with Respect to Gender					
PSS−10	Reference Values	Entire Group	Female Group	Male Group	Chi-Squared Test with Yates Correction			Fisher’s Exact Unilateral Test		Sample Size for 80% Test Power
		*n* = 50	*n* = 37	*n* = 13	Chi^2^	df	*p*-Value	*p*-Value	1 − β	*n*
					Low Stress vs. All Others					
Low stress	0–13	11 (22%)	8 (22%)	3 (23%)	0.079	1	0.779	0.596	0.029	56410
Moderate stress	14–26	33 (66%)	24 (65%)	9 (69%)						
High stress	27–40	6 (12%)	5 (14%)	1 (8%)						

% percentage within column (% entire group; % female group; % male group, respectively).

**Table 2:** Perceived Stress Scale with respect to the PSS−10 in the study group (*n* = 50) and for the group of females (*n* = 37) and males (*n* = 13). Mean standard deviation (±SD) and median (Me) are given.

	Reference Value	Entire Group *n* = 50			Female Group *n* = 37			Male Group *n* = 13			Mann–Whitney U Test		Sample Size for 80% Test Power
		Mean	±SD	Me	Mean	±SD	Me	Mean	±SD	Me	*p*-Value	1 − β	*n*
PSS−10	0–40	18.00	6.31	17.00	18.24	6.19	17.00	17.31	6.87	19.00	0.748	0.111	1665

**Table 3:** Levels of depression with respect to the BDI. Numerical and percentage values are given for the study group (*n* = 50) and for the group of females (*n* = 37) and males (*n* = 13).

					Comparison with Respect to Gender					
BDI	Reference Values	Entire Group	Female Group	Male Group	Chi-Squared Test with Yates Correction			Fisher’s Exact Unilateral Test		Sample Size for 80% Test Power
		*n* = 50	*n* = 37	*n* = 13	Chi^2^	df	*p*-Value	*p*-Value	1 − β	*n*
					Normal vs. All Others					
Normal	1–10	35 (70%)	27 (73%)	8 (62%)	0.178	1	0.673	0.330	0.950	480
Mild mood disturbance	11–16	9 (18%)	5 (14%)	4 (31%)						
Borderline clinical depression	17–20	3 (6%)	3 (8%)	0 (0%)						
Moderate depression	21–30	3 (6%)	2 (5%)	1 (8%)						
Severe depression	31–40	0 (0%)	0 (0%)	0 (0%)						
Extreme depression	>40	0 (0%)	0 (0%)	0 (0%)						

% percentage within column (% entire group; % female group; % male group, respectively).

**Table 4:** Level of depression with respect to the BDI questionnaire in the study group (*n* = 50) and for the group of females (*n* = 37) and males (*n* = 13). Mean standard deviation (±SD) and median (Me) are given.

	Reference Value	Entire Group *n* = 50			Female Group *n* = 37			Male Group *n* = 13			Mann–Whitney U Test		Sample Size for 80% Test Power
		Mean	±SD	Me	Mean	±SD	Me	Mean	±SD	Me	*p*-Value	1 − β	*n*
BDI	0–63	8.94	6.37	8.00	8.68	6.26	8.00	9.69	6.86	8.00	0.579	0.118	1430

**Table 5:** Neck disability with respect to the NDI. Numerical and percentage values are given for the study group (*n* = 50) and for the group of females (*n* = 37) and males (*n* = 13).

					Comparison with Respect to Gender					
NDI	Reference Values	Entire Group	Female Group	Male Group	Chi-Squared Test with Yates Correction			Fisher’s Exact Unilateral Test		Sample Size for 80% Test Power
		*n* = 50	*n* = 37	*n* = 13	Chi^2^	df	*p*-Value	*p*-Value	1 − β	*n*
					No Disability vs. All Others					
No disability	0–4	9 (18%)	7 (19%)	2 (15%)	0.018	1	0.893	0.571	0.033	2970
Mild	5–14	30 (60%)	22 (59%)	8 (62%)						
Moderate	15–24	10 (20%)	8 (22%)	2 (15%)						
Severe	25–34	1 (2%)	0 (0%)	1 (8%)						
Complete	>34	0 (0%)	0 (0%)	0 (0%)						

% percentage within column (% entire group; % female group; % male group, respectively).

**Table 6:** Cervical spine dysfunction with respect to the NDI questionnaire in the study group (*n* = 50) and for the group of females (*n* = 37) and males (*n* = 13). Mean standard deviation (±SD) and median (Me) are given.

	Reference Value	Entire Group *n* = 50			Female Group *n* = 37			Male Group *n* = 13			Mann–Whitney U test		Sample Size for 80% Test Power
		Mean	±SD	Me	Mean	±SD	Me	Mean	±SD	Me	*p*-Value	1 − β	*n*
NDI	0–50	10.16	6.27	9.00	9.57	5.95	9.00	11.85	7.10	12.00	0.308	0.272	280

**Table 7:** Multiple linear regression model with the PSS−10 as the dependent variable and BDI and NDI as independent variables.

	Regression Coefficient (b)	SE	Standardized Coefficient (β)	t-Value	*p*-Value	Tolerance Score	R^2^ Value	Semipartial Correlations (r)	Durbin-Watson Statistic
Intercept	10.007	1.289	-	7.760	0.000 *	-	-	-	
BDI	0.507	0.111	0.511	4.573	0.000 *	0.799	0.201	0.457	2.010
NDI	0.341	0.112	0.339	3.030	0.004 *	0.799	0.201	0.303	

* *p* < 0.05 statistical significance. R = 0.729; R^2^ = 0.531; Adjusted R^2^ = 0.511; F(2,47) = 26.600 *p* < 0.00). Standard error of the estimate: 4.416; SE—standard error.

## Data Availability

The article contains complete data used to support the findings of this study.
